# The rice SnRK family: biological roles and cell signaling modules

**DOI:** 10.3389/fpls.2023.1285485

**Published:** 2023-10-31

**Authors:** Seungmin Son, Sang Ryeol Park

**Affiliations:** National Institute of Agricultural Sciences, Rural Development Administration, Jeonju, Republic of Korea

**Keywords:** abiotic stress, biotic stress, cell signaling, phosphorylation, plant growth and development, rice, SNF1-related protein kinase

## Abstract

Stimulus-activated signaling pathways orchestrate cellular responses to control plant growth and development and mitigate the effects of adverse environmental conditions. During this process, signaling components are modulated by central regulators of various signal transduction pathways. Protein phosphorylation by kinases is one of the most important events transmitting signals downstream, via the posttranslational modification of signaling components. The plant serine and threonine kinase SNF1-related protein kinase (SnRK) family, which is classified into three subgroups, is highly conserved in plants. SnRKs participate in a wide range of signaling pathways and control cellular processes including plant growth and development and responses to abiotic and biotic stress. Recent notable discoveries have increased our understanding of how SnRKs control these various processes in rice (*Oryza sativa*). In this review, we summarize current knowledge of the roles of OsSnRK signaling pathways in plant growth, development, and stress responses and discuss recent insights. This review lays the foundation for further studies on SnRK signal transduction and for developing strategies to enhance stress tolerance in plants.

## Introduction

Plants play important roles in human survival by serving as energy sources and generating atmospheric oxygen. Humans have been cultivating crops since the Neolithic Age, which has contributed to human development by supplying stable, abundant sources of energy (Shaw et al., 2020). Therefore, the preservation and maintenance of plants are directly related to human life. However, the increasing worldwide population and industrial activities are causing global climate change. This is leading to changes in environmental factors (e.g., temperature, relative humidity, and salt concentrations in soil) essential for plant growth and development, resulting in enormous losses in crop yields (Parmesan and Hanley, 2015). In addition, climate change exacerbates plant diseases by creating environmental conditions that increase pathogen sensitivity and reduce plant immunity (Son and Park, 2022b). These challenges put global nutritional safety at extreme risk, and this problem is expected to become increasingly serious in the future. Although various advanced biotechnology tools have been developed (e.g., CRISPR/Cas9 [clustered regularly interspaced short palindromic repeats/CRISPR-associated nuclease 9], temporal-spatial gene expression, and translational control), these tools require the identification of suitable genes conferring desired traits and knowledge of their working mechanisms (Dutt et al., 2014; Son and Park, 2022a; Son and Park, 2023). Therefore, the exploration of key regulators associated with stress tolerance represents a key strategy to help mitigate the upcoming crisis through plant breeding.

**Figure 1:**
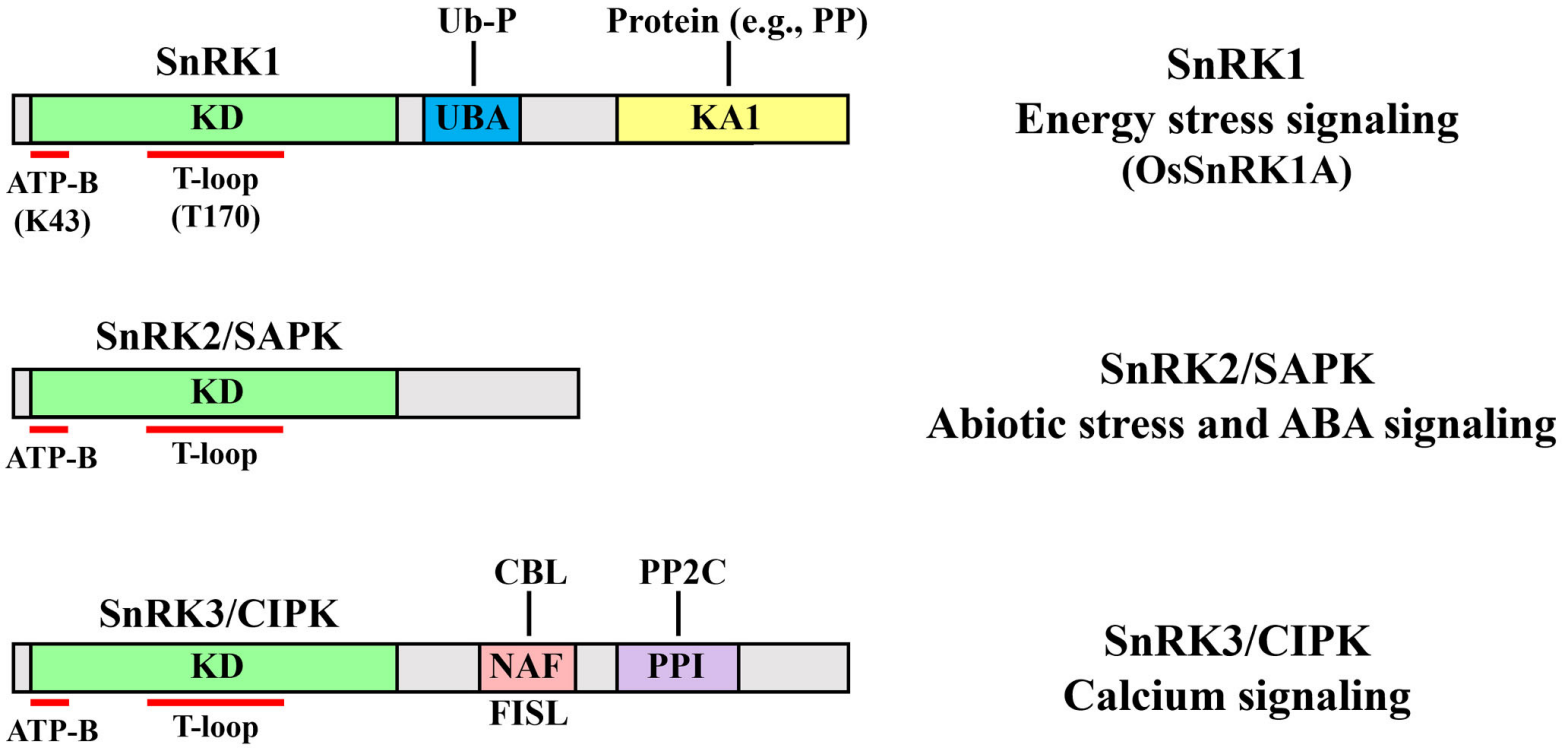
Structural domains and major functions of the three SnRK subfamilies. Top, SNF1-related protein kinase 1 (SnRK1) members contain a highly conserved N-terminal α-subunit kinase catalytic domain (KD) and a C-terminal regulatory domain including the ubiquitin-associated (UBA) and kinase-associated 1 (KA1) domains. Two amino acids in the KD are required for its kinase activity: the conserved lysine 43 (K43) conferring ATP binding (ATP-B) and the phosphorylated threonine 170 (T170) in the activation loop (T-loop) of OsSnRK1A are both required for its activation. The UBA domain interacts with the ubiquitination protein (Ub-P) and enhances its catalytic activity. The KA1 domain interacts with proteins including protein phosphatases (PPs). SnRK1s function as master regulators of energy-stress signaling. Middle, SnRK2/Stress-activated protein kinase (SAPK) members contain a KD and a divergent C-terminal domain and are central regulators of abiotic stress and ABA signaling. Bottom, SnRK3/Calcineurin B-like protein (CBL)-interacting protein kinase (CIPK) members contain a KD and a C-terminal regulatory domain containing a NAF or FISL motif (comprising 21 amino acids including the highly conserved [N, A, and F] or [F, I, S and L] residues) and a protein phosphatase interaction (PPI) domain. The autoinhibitory NAF or FISL motif interacts with CBL, resulting in CIPK activation via CBL-CIPK complex formation. The PPI domain interacts with the type 2C protein phosphatase (PP2C). Therefore, SnRK3s play important roles in the Ca^2+^ signaling pathway.

qwerThe catalytic subunit of the yeast serine and threonine protein kinase Sucrose non-fermenting 1 (SNF1) was first discovered in *Saccharomyces cerevisiae* (Carlson et al., 1981). SNF1, a heterotrimeric kinase consisting of one catalytic α-subunit and two noncatalytic subunits (such as β- and βγ-subunits), plays crucial roles in plant responses to various environmental stresses, including nutrient limitation (Hedbacker and Carlson, 2008). Its highly conserved orthologs, such as the mammalian AMP-activated protein kinase (AMPK) and plant SNF1-related protein kinase 1 (SnRK1), are also well-known master regulators of energy-stress signaling that play central roles in various biological processes (Polge and Thomas, 2007). Plants also possess a unique SnRK subfamily, classified into the SnRK2 and SnRK3 subgroups—also known as stress-activated protein kinases (SAPKs) and calcineurin B-like protein (CBL)-interacting protein kinases (CIPKs), respectively. These proteins, which are classified based on their domain composition, are mainly involved in various signaling pathways (**Figure 1**). SnRK1 proteins, consisting of a highly conserved N-terminal α-subunit kinase catalytic domain (KD) and a C-terminal regulatory domain containing a ubiquitin-associated domain and kinase-associated 1 domain, play central roles in energy-stress signaling (Emanuelle et al., 2015). SnRK2 proteins, containing a KD and a divergent C-terminal domain, play important roles in abiotic stress and abscisic acid (ABA) signaling (Kulik et al., 2011). SnRK3 proteins, which have a KD and a C-terminal regulatory domain containing an NAF or FISL motif and a protein-phosphatase interaction domain (and are thus known as CIPKs), play important roles in calcium (Ca^2+^) signaling (Hrabak et al., 2003).

Rice (*Oryza sativa*) is a staple food crop, providing nutrients and calories to approximately half the world’s population. The consumption of this crop is expected to continuously increase (Singh et al., 2018). However, rice yields are seriously damaged by abiotic and biotic stress globally, which will become more severe due to climate change. Fortunately, efficient new plant breeding techniques are universally available, including the use of site-directed nucleases (Schaart et al., 2016). These techniques are suitable for rice due to the availably of huge amounts of genomic resources and the small genome size and high transformation efficiency of this crop (Mishra et al., 2018). Therefore, it is crucial to identify genes conferring stress resilience and to unravel the underlying regulatory signaling pathways. Rice contains 47 *OsSnRK* genes, including 3 OsSnRK1s, 10 OsSnRK2s, and 34 OsSnRK3s. OsSnRKs play vital roles in regulating signaling pathways related to plant growth, development, and stress response. In this review, we provide an overview of the roles of OsSnRKs in rice with a focus on recent discoveries.

## OsSnRK1

SnRK1 is master regulators of energy-stress signaling that control various plant processes (Wurzinger et al., 2018; Jamsheer et al., 2021; Peixoto and Baena-Gonzalez, 2022). The rice SnRK1 subfamily comprises three members categorized into two subgroups: OsSnRK1A/OSK1 and OsSnRK1B (i.e., OSK24 and OSK35). The roles and the functional mechanisms of OsSnRK1s in plant growth, development, and stress responses are overviewed and discussed below.

## OsSnRK1 in plant growth

**Figure 2:**
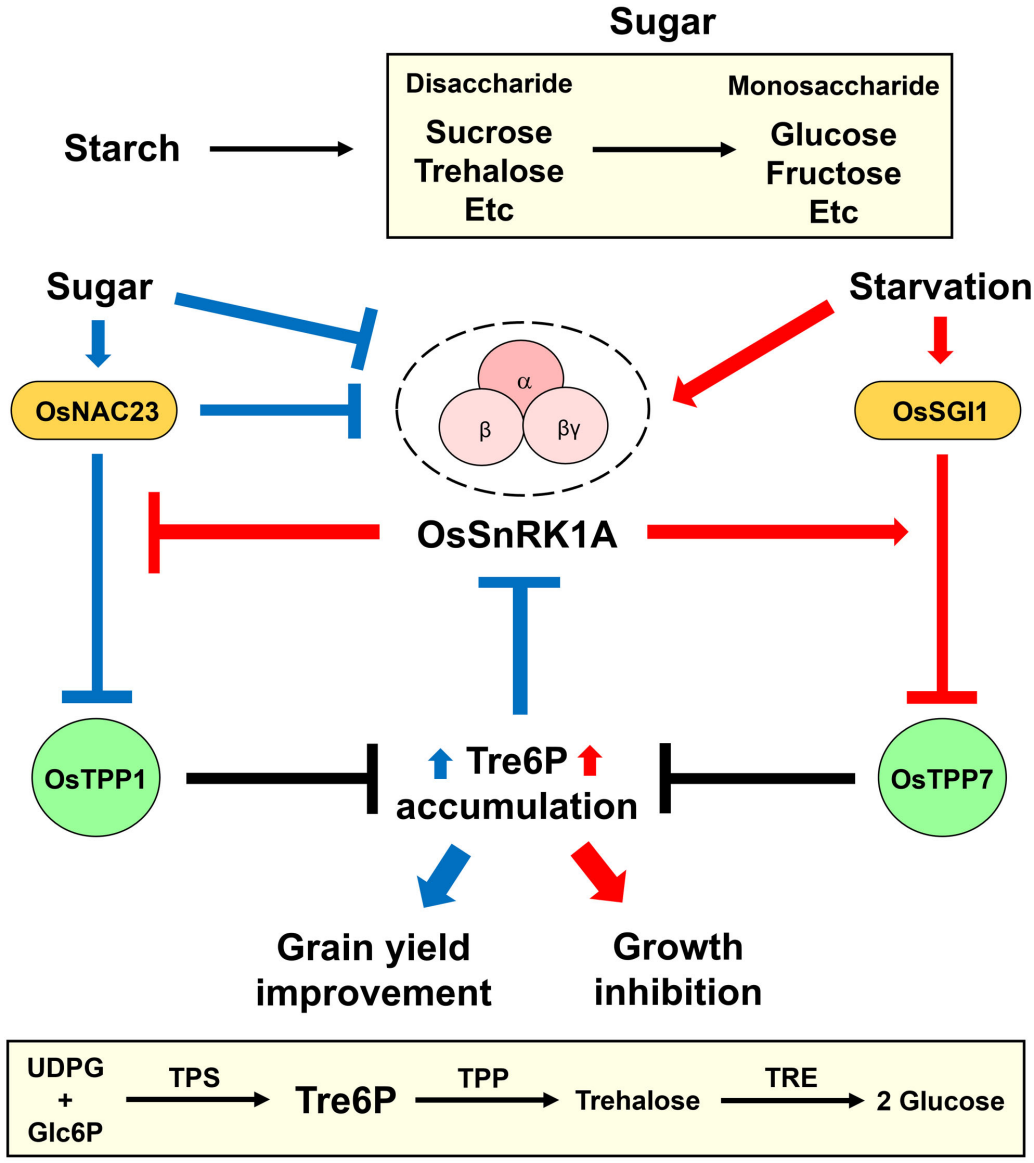
OsSnRK1A-Tre6P signaling loop functions in rice growth and development. The energy-stress master regulator SNF1-related protein kinase 1 (SnRK1) and Trehalose 6-phosphate (Tre6P) play central roles in plant energy homeostasis, growth, and development. Tre6P levels are very low in the basal state but respond dramatically to changes in sugar contents. Tre6P is produced from UDP-glucose (UDPG) and glucose-6-phosphate (Glc6P) by trehalose-6-phosphate synthase (TPS). Subsequently, Tre6P is converted to trehalose by trehalose-6-phosphate phosphatase (TPP) and is hydrolyzed into two glucose molecules by trehalase (TRE). Two groups recently revealed the roles of SnRK1-Tre6P signaling in regulating plant growth and development (Li et al., 2022; Wang et al., 2023). High sugar content increases the activity of the NAC (NAM, ATAF, and CUC) transcription factor OsNAC23 in repressing OsSnRK1A and Trehalose 6-phosphate phosphatase 1 (OsTPP1) activity (Li et al., 2022). Since TPPs convert Tre6P to trehalose, OsNAC23-mediated repression of OsTPP1 leads to Tre6P accumulation under high sugar levels. To maintain sugar homeostasis, the OsNAC23-induced Tre6P signal enhances the movement of sugar from source to sink, resulting in improved grain yield. Under energy-stress condition, OsSnRK1A and Starvation-associated growth inhibitor 1 (OsSGI1)/Basic helix–loop–helix 111 (OsbHLH111) are activated (Wang et al., 2023). OsSGI1 (when phosphorylated by OsSnRK1A) represses the expression of *OsTPP7* and increases Tre6P levels under sugar-deficiency condition. Hence, high Tre6P levels induced by the OsSnRK1A-OsSGI1-OsTPP7 module result in inhibited plant growth under sugar-starvation condition.

SnRK1 negatively regulates plant growth and development by affecting energy homeostasis and stress tolerance. SnRK1 induces genome-wide transcriptome reprogramming and metabolic changes, thereby inhibiting plant growth and development (Baena-Gonzalez et al., 2007; Cho et al., 2012; Belda-Palazón et al., 2020; Jamsheer et al., 2021). SnRK1 also plays an important role in Arabidopsis (*Arabidopsis thaliana*) meristem cells to inhibit plant growth in response to stress (Belda-Palazón et al., 2022; Son et al., 2023). Filipe et al. reported that the growth and development (e.g., flowering and seed yield) of rice plants were suppressed in 12-week-old *OsSnRK1A*-overexpressing transgenic plants compared to wild-type plants (Filipe et al., 2018). The regulatory mechanism of growth repression by OsSnRK1A was recently unraveled (Wang et al., 2023). Sugar starvation increases the expression of *Starvation-associated growth inhibitor 1*/*Basic helix–loop–helix 111* (*OsSGI1*/*OsbHLH111*), encoding a transcription factor that negatively regulates germination, growth, and agronomic traits (i.e., flowing time, grain length, grain width, and 1000-grain weight) (Wang et al., 2023; Yin et al., 2023). This transcription factor interacts with OsSnRK1A and regulates global gene expression, primarily of genes related to photosynthesis, mitogen-activated protein kinase (MAPK) signaling, and plant–pathogen interactions (Wang et al., 2023). Under sugar-deficiency condition, the interaction between OsSnRK1A and OsSGI1 increases, and OsSnRK1A phosphorylates OsSGI1 at serine 167 (Ser167). The OsSnRK1A-mediated OsSGI1 phosphorylation enforces the direct binding of OsSGI1 to the *Trehalose 6-phosphate phosphatase 7* (*OsTPP7*) promoter to repress its transcription (Wang et al., 2023). Since TPP converts trehalose 6-phosphate (Tre6P/T6P) to trehalose, the reduced expression of various *OsTPP* genes (e.g., *OsTPP7*) by OsSGI1 increases the Tre6P content but decreases the sucrose content in rice. Therefore, the OsSnRK1A-OsSGI1-OsTPP7 module represses plant growth under sugar-starvation condition (**Figure 2**).

Tre6P inhibits SnRK1 activity and plays dual roles as a signaling molecule and a homeostatic regulator of plant growth and development (Fichtner and Lunn, 2021). Tre6P level is normally very low in plants, but it is altered dramatically in response to changes in sucrose contents (Yadav et al., 2014; Peixoto et al., 2021). Trehalose-6-phosphate synthase (TPS) generates Tre6P using UDP-glucose (UDPG) and glucose-6-phosphate (Glc6P). Trehalose-6-phosphate phosphatase (TPP) then converts Tre6P to trehalose, which is hydrolyzed into two glucose molecules by trehalase (TRE) (**Figure 2**). Although Tre6P is a vital signaling molecule for plant growth and development (Zhang et al., 2009; Nunes et al., 2013; Zhai et al., 2018), Arabidopsis growth was repressed by high levels of Tre6P accumulation (Schluepmann et al., 2003; Schluepmann et al., 2004). Thus, the OsSnRK1A-OsSGI1-OsTPP7 module increases Tre6P levels, which is thought to inhibit plant growth under sugar-starvation conditions. Interestingly, the phosphorylation of OsSGI1 by OsSnRK1A also leads to OsSGI1 degradation under sugar-deficiency conditions (Wang et al., 2023). The authors suggested that this effect is related to the toxicity of OsSGI1, suggesting that OsSnRK1A helps maintain OsSGI1 at manageable levels. However, the detailed mechanisms and signaling components involved in OsSnRK1-signaling-regulated plant growth still need to be elucidated.

## OsSnRK1 in plant development

Many studies of the biological functions of OsSnRK1A indicate that it plays central roles in the overall plant lifecycle, from germination to grain development. Germination and seedling development from the embryo are heterotrophic processes by which the plant gains energy and resources from the source tissue, endosperm (Graham, 2008; Quettier and Eastmond, 2009; Tan-Wilson and Wilson, 2012). Therefore, resource mobilization from the endosperm to sink tissues is important for germination and seedling establishment.

Lu et al. showed that OsSnRK1A is a key regulator of germination and seedling establishment in rice (Lu et al., 2007). Starch comprises approximately 60–70% of raw grain weight in cereals, and it largely affects the food quality of rice (Wang et al., 2021). The starch-metabolizing enzyme α-amylase plays a central role in the mobilization of starch during germination and seedling development. α-Amylase 3 (αAmy3)/RAmy3D/Amy3D and αAmy8/RAmy3E/Amy3E are major amylases that regulate germination and seedling development in rice by hydrolyzing starch into sugars, which serve as a carbon source under energy deficiency conditions (Yu et al., 1996; Chen et al., 2006). Molecular and genetic data showed that OsSnRK1A transactivates the promoter of *V-myb avian myeloblastosis viral oncogene homolog S1* (*MYBS1*), encoding a transcription factor that directly induces the expression of *αAmy3*, and this module is required for rice grain germination and seedling establishment (Lu et al., 2007).

The expression of α-amylase genes is also regulated by the phytohormone gibberellin (GA), which is synthesized in the embryo of the germinating rice grain and diffuses to the aleurone layer through the scutellum (Damaris et al., 2019). The GA-inducible transcription factor GAMYB/MYBGA directly binds to and activates the promoters of α-amylase genes in response to GA (Gubler et al., 1997). Glucose inhibits OsSnRK1A activity and the localization of MYBS1 to the nucleus (Lu et al., 2007; Hong et al., 2012). However, GA overcomes this effect via an interaction between MYBS1 and GAMYB, resulting in the nuclear localization of these transcription factors and the formation of a stable bipartite MYB-DNA complex (Hong et al., 2012). Therefore, the coordination of starvation-OsSnRK1A-MYBS1 and GA-GAMYB signaling determines the expression levels of genes (including α-amylase genes) required for germination and seedling establishment. In addition, Lin et al. showed that OsSnRK1A plays important role in source-sink communication controlling nutrient mobilization from source tissue (endosperm) to sink tissue (the germinating embryo and seedling) via the induction of α-amylase and other hydrolases (Lin et al., 2014). The authors also suggested that ABA promotes the interaction between OsSnRK1A and SnRK1A-interacting negative regulator 1/OXS3-like 8 (SKIN1/O3L8) and SKIN2 in the cytoplasm to inhibit the localization of SnRK1A and MYBS1 to the nucleus under abiotic stress. Moreover, Small auxin-up RNA 33 (OsSAUR33), which enhances rice grain vigor by regulating resource mobilization, was identified as a binding partner of OsSnRK1A (Zhao et al., 2021). These findings suggest that OsSnRK1A controls resource mobilization to promote germination and early seedling growth for seedling establishment in rice. However, the roles and regulatory mechanisms of SnRK1 during this early stage are complex and poorly understood.

OsSnRK1A and Tre6P signaling are important for rice grain development. *OsTPS8* transcript levels and Tre6P levels increased in inferior spikelets of large panicle rice plants (varieties CJ03 and W1844) when the upper two-thirds of spikelets were removed, while *OsSnRK1A* transcript levels decreased (Jiang et al., 2021). Based on this observation, the authors suggested that crosstalk between OsSnRK1A and Tre6P signaling regulates sucrose metabolism to initiate inferior grain filling in rice. The key signaling components and regulatory mechanism of the OsSnRK1A-Tre6P signaling pathway were recently reported (**Figure 2**). NAC (NAM, ATAF, and CUC) transcription factors play various roles in plant growth, development, and stress responses (Singh et al., 2021). OsNAC23 is thought to regulate rice grain size due to its specific and dramatic effects on gene expression during seed development (Mathew et al., 2016). Li et al. proposed that OsNAC23 is a sugar sensor due to the positive correlation between OsNAC23 levels (i.e., mRNA and protein) and sugar content (Li et al., 2022). OsNAC23 increases rice grain yield and Tre6P content, while it decreases sugar (including trehalose) content (Li et al., 2022). Molecular and genetic data indicate that OsNAC23 directly binds to the promoter of *OsTPP1*, reducing its expression and inhibiting Tre6P-to-trehalose conversion, resulting in high Tre6P levels and low sugar levels. Notably, OsSnRK1A and OsNAC23 inhibit each other’s activity (Li et al., 2022). OsSnRK1A represses OsNAC23 at the posttranslational and transcriptional levels via direct protein phosphorylation and indirectly reducing its transcript levels. In addition, OsNAC23 indirectly suppresses *OsSnRK1A* transcription. Therefore, the OsNAC23-mediated Tre6P accumulation by repressing OsSnRK1A and OsTPP1 improved grain yield under high sugar levels (**Figure 2**). To maintain sugar homeostasis, Tre6P functions as a negative feedback regulator, decreasing sugar content to maintain its basal levels in response to high sugar content (Figueroa and Lunn, 2016). The authors suggested that a feed-forward regulatory loop consisting of OsNAC23, Tre6P, and OsSnRK1A helps maintain sugar homeostasis and grain yield (Li et al., 2022).

Expression profiling suggested that OsSnRK1s perform distinct roles in rice (Takano et al., 1998). Among OsSnRK1B subfamily members, OSK24 is thought to be involved in carbohydrate metabolism during the development of sink tissues including the caryopsis (Kanegae et al., 2005). However, to date, for OsSnRK1B subfamily members, only the role and signaling pathway of these proteins in regulating photoperiodic flowering have been described in detail. Rice is a short-day plant whose flowing regulatory mechanism is well described in an earlier review (Brambilla and Fornara, 2013). Heading date repressor 1 (HDR1) directly interacts with OSK24 in the nucleus and delays flowering time under long-day conditions (Sun et al., 2016b). *OSK24*-silenced rice plants showed early flowering and expression patterns of flowering genes similar to those of the *hdr1* mutant. HDR1-OSK24 nuclear complex indirectly induces the transcription of *Heading date 1* (*Hd1*) and reduces the transcription of *Early heading date 1* (*Ehd1*), while it directly phosphorylates HD1 protein (Sun et al., 2016b). However, the effect of HD1 phosphorylation by OSK24 is not yet known.

## OsSnRK1 in plant response to abiotic stresses

Among abiotic stresses, the role of OsSnRK1 signaling has been best studied during flooding. Flooding causes energy stress in plants due to impaired photosynthesis and respiration; thus, plants experiencing flooding redistribute their energy source by inhibiting anabolic processes and inducing catabolic processes. Overexpressing *OsSnRK1A* enhanced stress tolerance in Arabidopsis under submerged energy-deficiency conditions by regulating stress-inducible gene expression (Cho et al., 2012). In addition, Arabidopsis global protein translation, whose progress must be suppressed to conserve energy under flooding conditions, is downregulated by OsSnRK1A (Son et al., 2022). Rice has developed not only the quiescence strategy but also the escape strategy (Voesenek and Bailey-Serres, 2009). OsCIPK15-upregulated OsSnRK1A drives energy production and coleoptile elongation to escape flooding condition (Lee et al., 2009). OsTTP7, the genetic determinant in the major quantitative trait locus (QTL) *qAG-9-2* (related to flooding tolerance during germination), prevents Tre6P accumulation to activate OsSnRK1A, thereby enhancing germination and coleoptile elongation under submergence conditions (Kretzschmar et al., 2015). Conversely, SKIN1 and SKIN2, which negatively regulate OsSnRK1A activity, reduce seedling growth by impairing sugar production under submergence-mediated hypoxia conditions (Lin et al., 2014). Submergence-induced FCS-like zinc finger 18 (OsFLZ18) directly binds to OsSnRK1A and negatively regulates the OsSnRK1A-mediated induction of *αAmy3*, inhibiting coleoptile elongation when germinated and grown underwater (Ma et al., 2021). The key OsCIPK15-OsSnRK1A-MYBS1 signaling pathway conferring anaerobic germination tolerance is discussed in the sector related to OsSnRK3/OsCIPK below.

OsSnRK1s are also involved in plant responses to other abiotic stresses, including drought and cold. Drought and cold markedly induce the transcription of *SKIN* genes (Lin et al., 2014). ABA, a major phytohormone involved in abiotic stress responses, promotes SKINs-OsSnRK1A interactions in the cytoplasm, preventing the nuclear localization of OsSnRK1A with MYBS1 (Lin et al., 2014). In addition, the transcript levels and activities of OsSnRK1s (OsSnRK1A, OSK24, and OSK35) increased in *C4 phosphoenolpyruvate carboxylase*-expressing transgenic rice plants conferring enhanced drought tolerance (Liu et al., 2017). The expression levels of *SnRK1* genes were also higher in cold-tolerant rice variety ZZ39 than in the cold-susceptible variety RIL82 under cold stress (Yu et al., 2020). However, the relevant signaling components and regulatory mechanisms in rice are unknown.

## OsSnRK1 in plant response to biotic stresses

OsSnRK1s also play important roles in innate immunity against various pathogens. Overexpressing *OsSnRK1A* enhanced immunity against various pathogens (i.e., *Xanthomonas oryzae* pv. *oryzae* [*Xoo*], *Magnaporthe oryzae*, *Cochliobolus miyabeanus*, and *Rhizoctonia solani*) in rice, while *OsSnRK1A* silencing reduced this immunity (Filipe et al., 2018). Comparative proteome profiling revealed many *Xoo*-responsive proteins in susceptible (Dongjin) and resistant (Hwayeong) rice cultivars, and interactome analysis showed that some Hwayeong-specific *Xoo*-responsive proteins are OsSnRK1A binding proteins (Gupta et al., 2022). As with *OsSnRK1A*, overexpressing *OSK35* also increased rice immunity against *Xoo* and *M. oryzae*, whereas this immunity was impaired in the *osk35* mutant (Kim et al., 2015a).

Interestingly, two recent studies unveiled the detailed regulatory mechanism of OsSnRK1A in rice immunity. Plants have two immune systems known as pathogen-associated molecular pattern (PAMP)-triggered immunity (PTI) and effector-triggered immunity (ETI). The *Xanthomonas* resistance gene *XA21* encodes a receptor kinase localized to the plasma membrane (PM) and endoplasmic reticulum (ER) (Chen et al., 2010a; Park et al., 2010). XA21 recognizes the sulfated peptide RaxX/Ax21 of the bacterial pathogen *Xoo* to trigger PTI; thus, XA21 confers broad-spectrum resistance to different *Xoo* strains (Ercoli et al., 2022). The ATPase activity of XA21-binding protein 24 (XB24) induces the autophosphorylation of XA21 to inhibit XA21-mediated immune responses when the defense response does not need to be activated (Chen et al., 2010b). Seo et al. identified OsSnRK1A as the XB24 binding partner that activates XA21-mediated immunity based on interactome and systems analyses (Seo et al., 2011). However, the regulatory mechanism of OsSnRK1A in PTI has been elusive. Yang et al. recently showed that XB24 is required for PTI against fungal pathogens (i.e., *M. oryzae* and *Ustilaginoidea virens*) in a non-*XA21* rice variety (Yang et al., 2022a). PAMP-activated OsSnRK1A directly interacts with and phosphorylates cytosolic XB24 at threonine 83 (Thr83) to increase its ATPase activity, resulting in the initiation of pathogenesis-related (*PR*) gene expression and an oxidative burst (Yang et al., 2022a). This defense signaling is impaired by Small cysteine-rich effector 1 (SCRE1) of *U. virens*. SCRE1 directly binds to XB24 and represses its ATPase activity by inhibiting OsSnRK1A-mediated phosphorylation as well as ATP binding (Yang et al., 2022a).

The factors that function upstream of OsSnRK1A signaling in rice immunity were also recently revealed. Ubiquitination-mediated posttranslational modification plays a key role in plant immunity signaling (Gao et al., 2022b). This process is mediated by three enzymes: a ubiquitin-activating enzyme (UBA/E1), ubiquitin-conjugating enzyme (UBC/E2), and E3 ubiquitin ligase (Callis, 2014). Liu et al. recently determined that innate immunity against pathogens, including *M. oryzae*, and OsSnRK1A activity were both increased in *OsUBC13*-silenced rice plants (Liu et al., 2023). OsUBC13 directly interacts with OsSnRK1A and contributes to lysine 63 (K63)-linked polyubiquitination of this kinase, leading to the inactivation of OsSnRK1A without affecting its protein stability (Liu et al., 2023). Therefore, OsUBC13-mediated OsSnRK1A ubiquitination inhibits rice immunity against *M. oryzae*. By contrast, the rice deubiquitinating enzyme Otubain 1.1 (OsOTUB1.1) confers resistance to *M. oryzae* by directly interacting with OsSnRK1A and attenuating its K63-linked polyubiquitination (Liu et al., 2023).

## OsSnRK2/OsSAPK

SnRK2s are plant-specific kinases that primarily function in abiotic stress and ABA signaling. The biological roles and signaling pathways of SnRK2s are well studied in Arabidopsis (Kulik et al., 2011; Maszkowska et al., 2021; Hasan et al., 2022). ABA is a major phytohormone regulating various abiotic stress responses, and SnRK2 plays pivotal roles in ABA signaling pathways. In the absence of ABA, type 2C protein phosphatases (PP2Cs) including Abscisic acid insensitive 1 (ABI1) inactivate SnRK2s (Ali et al., 2020). However, in the presence of ABA, the ABA receptor PYR/PYL/RCAR (Pyrabactin-resistance/Pyrabactin-resistance like/Regulatory component of ABA receptors) perceives ABA and inhibits PP2Cs to activate SnRK2s (Song et al., 2022). SnRK2-activated ABA signaling induces massive changes in gene expression by controlling the binding of transcription factors including Basic leucine zippers (bZIPs) to ABA-responsive elements (ABRE; PyACGTGGC, where Py is a pyrimidine base [C or T]) in their target genes (Joo et al., 2021). In addition, PYR/PYL/RCAR regulates the activities of the outward Slow anion channels (SLACs), which regulate stomatal movement, a process critical for enhancing drought tolerance (Assmann and Jegla, 2016).

**Figure 3:**
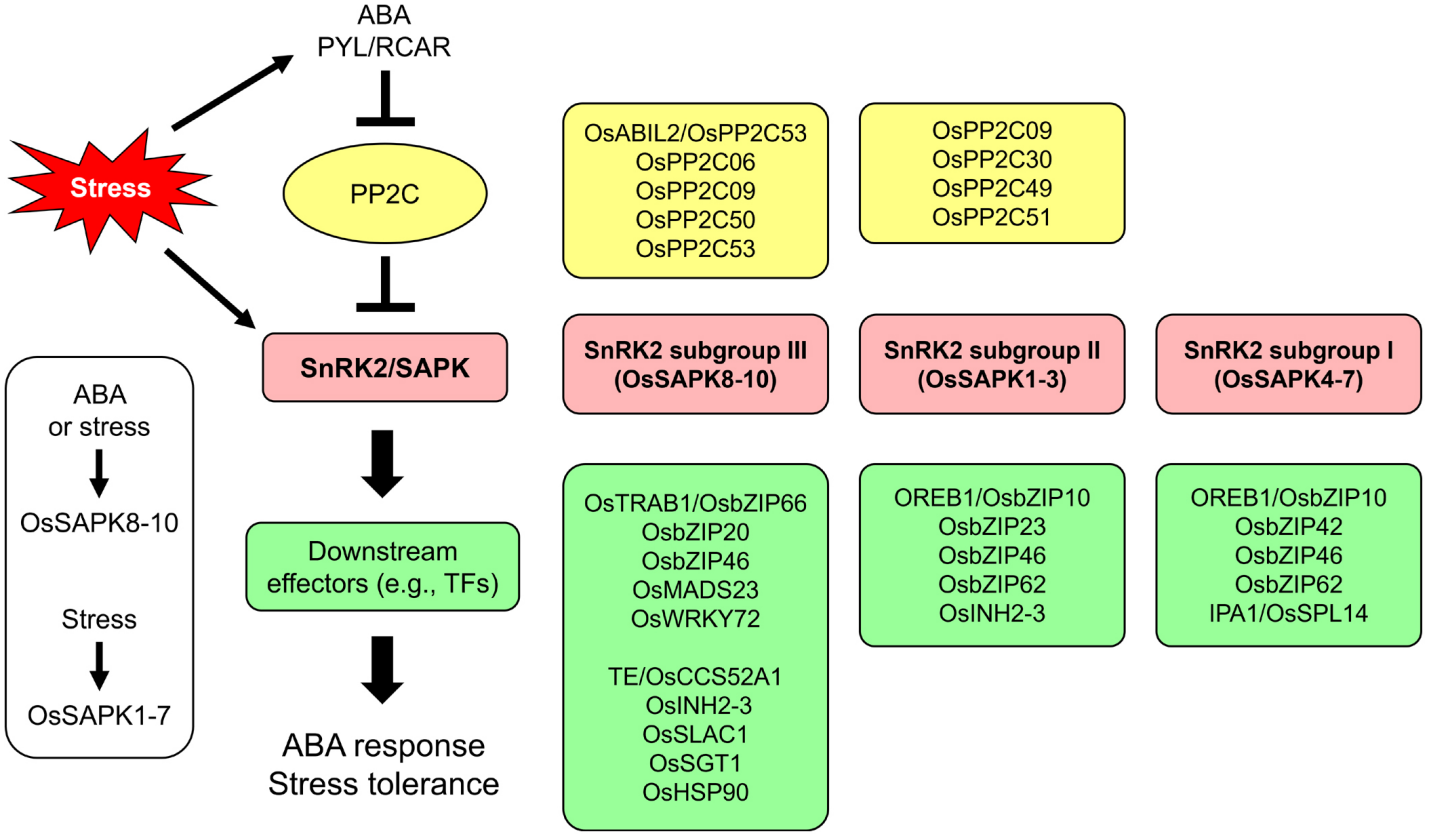
ABA signaling cascade and signaling components associated with OsSnRK2. SNF1-related protein kinase 2 (SnRK2)/Stress-activated protein kinase (SAPK) is a core regulator of ABA signaling and abiotic stress responses in plants. For the ABA response, the ABA receptor PYL/RCAR (Pyrabactin-resistance like/Regulatory component of ABA receptors) inactivates the type 2C protein phosphatase (PP2C), which is a negative regulator of SnRK2. Activated SnRK2 phosphorylates effectors including transcription factors to transfer the signal downstream. In rice, all OsSAPKs are activated by hyperosmotic stress, but only OsSAPK8–10 (belonging to subgroup III) are activated by ABA (Kobayashi et al., 2004). Many studies have identified various regulators of OsSAPK-mediated ABA signaling. This figure shows the signaling components known to be associated with the different OsSnRK2 subgroups.

The rice genome encodes 10 OsSnRK2 subfamily members, denoted Stress-activated protein kinase 1-10 (OsSAPK1–10), which are classified into three subgroups: Subgroup I (OsSAPK4–7), subgroup II (OsSAPK1–3), and subgroup III (OsSAPK8–10) (Kobayashi et al., 2004). The functions and regulation mechanisms of SnRK2s are well-conserved in rice. The signaling components and signaling pathways are summarized below (**Figure 3**).

## OsSnRK2 subgroup III in abiotic stress and ABA signaling

The transcript levels of *OsSAPK* genes change in response to abiotic stress and ABA (Kobayashi et al., 2004; Yu et al., 2022). Since the functions of kinases depend on their enzyme activities, these activities are generally more important than transcript or protein abundance. Kobayashi et al. demonstrated that hyperosmotic stress induces the kinase activities of all OsSAPKs, but ABA treatment activates only the activities of subgroup III members OsSAPK8-10 (Kobayashi et al., 2004). The ABA-activated subgroup III members OsSAPK8–10 are the best-studied OsSnRK2s. OsSAPK8–10 phosphorylate OsTRAB1/OsABF5/OsbZIP66 (Transcription factors responsible for ABA regulation 1/ABRE-binding factor 5/Basic leucine zipper 66), which activate the promoters of their target genes containing ABREs, suggesting that OsSAPK8–10 regulate the ABA signaling pathway via the posttranslational modification of transcription factors via phosphorylation (Kobayashi et al., 2005). Much is known about the relationship between ABA-activated OsSAPK9 and transcription factors. Three OsSAPKs (OsSAPK2, OsSAPK6, and OsSAPK9) were identified as binding partners of OsbZIP46, as their constitutive activation via deletion of the autoinhibitory D domain resulted in enhanced tolerance to drought and osmotic stress (Tang et al., 2012). The OsPYL (i.e., RCAR2 and RCAR5)-OsPP2C06-OsSAPK9-OsbZIP46 signaling cascade was reconstituted in protoplasts (Kim et al., 2015b), and Tang et al. showed that OsSAPK9 promotes the transcriptional activity of OsbZIP46. This process is inhibited by Mediator of OsbZIP46 deactivation and degradation (MODD), which binds to the D domain of OsZIP46 (Tang et al., 2016). Moreover, the ABA-activated OsSAPK9-OsbZIP20 module enhances plant tolerance to high ammonium stress via ammonium assimilation, antioxidant defense, and the induction of *Late embryogenesis abundant* (*OsLEA*) genes (Sun et al., 2020). The MADS-domain transcription factor OsMADS23 directly activates the promoters of ABA- and jasmonic acid (JA)-biosynthetic genes to enhance phytohormone biosynthesis (Li et al., 2021; Lv et al., 2022). The rice E3 ubiquitin ligase Plant U-box protein 16 (OsPUB16) negatively regulates OsMADS23 via ubiquitination-mediated protein degradation (Lv et al., 2022). However, OsSAPK9-mediated OsMADS23 phosphorylation inhibits this process and upregulates the transcriptional activity of OsMADS23, thereby increasing drought and salt tolerance by enhancing ABA and JA biosynthesis (Li et al., 2021; Lv et al., 2022).

The conservation of PYL-PP2C-SnRK2 signaling in rice has been demonstrated for subgroup III OsSAPKs (**Figure 3**). The rice PP2C OsABI-like2 (OsABIL2)/OsPP2C53, a protein phosphatase that negatively regulates ABA signaling, interacts not only with the ABA receptor OsPYL/RCAR10 but also with OsSAPKs (i.e., OsSAPK8 and OsSAPK10). ABA-activated OsPYL/RCAR10 inhibits the activity of OsABIL2, which dephosphorylates and inactivates OsSAPK8 and OsSAPK10 (Li et al., 2015). *OsABIL2*-overexpressing transgenic rice plants showed decreased root hair elongation and salt sensitivity of the root meristem (which are positively related to ABA), whereas overexpressing *OsSAPK10* enhanced these phenotypes (Wang et al., 2017; Huang et al., 2021a). Furthermore, Han et al. showed that OsPP2C50 interacts with both OsPYL/RCAR3 and OsSAPK10 through the VxGΦL motif and dephosphorylates OsSAPK10 (Han et al., 2017). Mutating this motif modulated ABA signaling in rice protoplasts and Arabidopsis.

OsPP2C09, which interacts with OsSAPKs (OsSAPK1–2 and OsSAPK8–10) and various OsPYLs, also dephosphorylates OsSAPKs and represses ABA signaling (Miao et al., 2020). ABA-activated OsSAPK8, OsSAPK9, and OsSAPK10 interact with and phosphorylate Tiller enhancer (TE)/OsCCS52A1, which activates the E3 ligase Anaphase promoting complex/Cyclosome (APC/C) (Lin et al., 2015). OsSAPK-mediated TE phosphorylation impairs APC/C^TE^ activity by degrading the ABA receptor OsPYL/RCARs including RCAR10, whereas GA induces OsSAPK degradation by APC/C^TE^ (Lin et al., 2015). Rice Inhibitor 2 (OsINH2) and OsINH3, the regulatory subunits of the Protein phosphatase 1 complex, physically interact with the Type 1 protein phosphatases (OsTOPPs; OsTOPP1–5) and with OsSAPKs (i.e., OsSAPK1–3, OsSAPK8, and OsSAPK9) and negatively regulate ABA-mediated growth, development, and oxidative stress responses (Jadoon et al., 2022). TOPP1 and INH2/AtI-2 synergically reduce ABA signaling in Arabidopsis by inactivating OsSnRK2s, including OsSnRK2.6 (Hou et al., 2016). Thus, the action of the TOPP complex-OsSnRK2 in ABA signaling is thought to be a general mechanism in plants.

The role of SnRK2 in ABA signaling associated with stomatal movement has also been elucidated. OsSAPK8 and OsSAPK10 directly interact with and phosphorylate the nitrate-selective S-type anion channel OsSLAC1, which is specifically expressed in guard cells (Sun et al., 2016a; Min et al., 2019). Furthermore, OsPP2C50 and OsPP2C53 (which are mainly expressed in guard cells) interact with both OsSAPK10 and OsSLAC1 to repress stomatal closure (Min et al., 2019). These findings indicate that the PP2C-SnRK2-SLAC module is highly conserved in Arabidopsis and rice (**Figure 3**).

## OsSnRK2 subgroup II in abiotic stress and ABA signaling

OsSnRK2 subgroup II comprises OsSAPK1–3. These proteins also interact with various signaling components involved in plant responses to abiotic stress and ABA (**Figure 3**). As mentioned above, OsSAPK1-3 were identified as binding partners of OsbZIP46, OsbZIP62, OsPP2C09, OsINH2, and OsINH3, respectively (Tang et al., 2012; Miao et al., 2020; Jadoon et al., 2022), but the detailed mechanisms are unknown. OsSAPK1, OsSAPK2, and OsSAPK6 were also identified as interacting partners of OsbZIP62, which positively regulates plant tolerance to drought and oxidative stress (Yang et al., 2019). The absence of the C-terminus of OsbZIP62 is necessary for its transcriptional activity, and OsbZIP62 interacts with three OsSAPKs, suggesting that the posttranslational modification of OsbZIP62 by OsSAPKs is required for its activity (Yang et al., 2019). However, the underlying mechanism has not yet been elucidated. Studies exploring the function and signaling pathway of OsSAPK1 have not been reported. Lou et al. recently suggested that OsSAPK3 increases drought tolerance in both ABA-dependent and ABA-independent manners, as well as increasing grain yield by upregulating the expression of genes related to nitrate transport and seed size (Lou et al., 2023). However, the regulatory mechanisms and signaling components involved are largely elusive.

Unlike OsSAPK1 and OsSAPK3, the functions and regulatory mechanisms of OsSAPK2 have been determined. Kim et al. identified the role of the highly conserved ABA signaling pathway consisting of OsPYL/RCAR5-OsPP2C30-OsSAPK2-OREB1/OsbZIP10 in ABA-mediated gene regulation in rice (Kim et al., 2012). OsPP2C51, which is a positive regulator of seed germination and interacts with OsPYL/RCAR5, OsSAPK2, and OREB1, dephosphorylated OREB1 (which had been phosphorylated by OsSAPK2) *in vitro* (Bhatnagar et al., 2017). Among OsSAPK subfamily members (i.e., OsSAPK2, OsSAPK6, and OsSAPK9), OsSAPK2 primarily induced the *trans*-activation activity of OsbZIP46 in rice protoplasts (Kim et al., 2015b). In addition, Zong et al. showed that OsSAPK2 phosphorylates and activates OsbZIP23, a key transcription factor regulating ABA signaling and drought tolerance in rice (Zong et al., 2016). Notably, OsSAPK2-activated OsbZIP23 directly induces the expression of *OsPP2C49*, which in turn inactivates OsSAPK2, representing a type of feedback regulation (Zong et al., 2016). CRISPR/Cas9-generated *ossapk2* mutants were insensitive to ABA but sensitive to abiotic stresses including drought (Lou et al., 2017). Stress-related genes and genes encoding antioxidant enzymes were downregulated in the *ossapk2* mutants, suggesting that OsSAPK2 increases drought tolerance via ABA signaling and its effect on the antioxidant defense system (Lou et al., 2017). *OsSAPK2* was also upregulated in the *Rolled and erect leaf 1* (*REL1*)-overexpressing *rel1-D* mutant, conferring leaf rolling, drought tolerance, senescence, and ABA responses (Liang et al., 2018). In summary, OsSnRK2 subgroup II members are thought to be mainly involved in the OsPYL/RCAR-OsPP2C-OsSAPK2 signaling cascade for ABA-mediated stress responses.

## OsSnRK2 subgroup I in abiotic stress and ABA signaling

OsSnRK2 subgroup I consists of four members OsSAPK4-7. Some downstream targets of these kinases are known, but their upstream regulators are largely elusive (**Figure 3**). Therefore, we will focus on the downstream signaling of this subgroup. OsSAPK6 interacts with the transcription factor OREB1 (Chae et al., 2007; Ding et al., 2009). OsSAPK6 phosphorylated OREB1 at multiple sites *in vitro*, and mutating three serine residues (Ser43, Ser44, and Ser47) significantly reduced OsSAPK6-induced OREB1 phosphorylation (Chae et al., 2007). However, the ectopic expression of *OsSAPK6* slightly reduced ABA sensitivity in tobacco, pointing to the interference of SnRK2 signaling due to the strong expression of *OsSAPK6* (Chae et al., 2007). Tang et al. showed that OsSAPKs including OsSAPK6 physically interact with OsbZIP46 and that OsbZIP46CA1, a constitutively active form of OsbZIP46, improves drought and osmotic stress tolerance (Tang et al., 2012). Co-expressing *OsSAPK6* and *OsbZIP46CA1* significantly increased ABA sensitivity and enhanced tolerance to drought, heat, and cold in rice (Chang et al., 2017). In addition, OsSAPK6 interacts with OsbZIP62, conferring drought tolerance and antioxidants (Yang et al., 2019). However, the underlying mechanism is unknown. A mass-spectrometry-based phosphoproteomic study showed that OsSAPK6 is phosphorylated by ABA (Qiu et al., 2017). The phosphorylation status of SnRK2s determines their activity, suggesting that OsSAPK6 can also be activated by ABA.

OsSAPK6 also plays a crucial role for cold tolerance in rice. The rice transcription factor IPA1/WFP/OsSPL14 (Ideal plant architecture 1/Wealthy farmer’s panicle/Squamosa promoter binding protein-like 14) is an invaluable regulator conferring not only grain yield but also broad-spectrum disease resistance (Wang et al., 2018a; Wang et al., 2018b; Liu et al., 2019). OsSAPK6 phosphorylates Ser201 and Ser213 of IPA1, resulting in its improved stability, and induces the expression of *IPA1* (Jia et al., 2022). The phosphorylated IPA1 directly activates the promoter of *C-repeat binding factor 3* (*OsCBF3*), encoding a dehydration-responsive element-binding (DREB) protein, thereby increasing the transcript abundance of cold-responsive genes (Jia et al., 2022). Genetic analysis also revealed that the chilling-induced OsSAPK6-IPA1-OsCBF3 signaling pathway is important for determining rice grain yield as well as cold tolerance (Jia et al., 2022).

OsSAPK4 controls ionic homeostasis, photosynthetic activity, and oxidative stress responses by regulating the expression of various genes, thereby improving salinity tolerance (Diedhiou et al., 2008). OsSAPK4 was identified as a binding partner of OREB1 and OsbZIP42 (Ding et al., 2009; Joo et al., 2021). Although OsSAPK4 did not interact with full-length OsbZIP42, which increases drought tolerance via ABA signaling, it interacted with a truncated version of OsbZIP42 lacking the D domain (Joo et al., 2019). OsbZIP42 was predicted to contain very short α-helical structures unlike other OsbZIPs, which contain several well-developed long α-helical structures, suggesting that additional modifications and/or conformational changes are needed for its interaction with OsSAPK4 (Joo et al., 2019). The roles and signaling components of OsSAPK5 and OsSAPK7 remain to be elucidated.

## OsSnRK2 in plant response to biotic stresses

Interesting, OsSnRK2s are also involved in plant responses to biotic stress. The transcript levels of *OsSAPK3*, *OsSAPK5*, *OsSAPK7*, and *OsSAPK9* significantly increased in response to *Xanthomonas oryzae* pv. *oryzicola* (*Xoc*) in a resistant rice variety (Xu et al., 2013). In addition, *OsSAPK9* and *OsSAPK10* transcript levels increased in response to *Rhizoctonia solani* in a resistant rice variety (Yang et al., 2022b). Zhang et al. showed that OsSAPK9, which forms a complex with the chaperones Suppressor of the G2 allele of SKP1 (OsSGT1) and Heat shock protein 90 (OsHSP90), regulates the expression of various defense-related genes and confers salt tolerance as well as resistance to *Xoo* (Zhang et al., 2019b). OsSAPK10 phosphorylates the Thr129 residue in OsWRKY72, which inhibits JA biosynthesis by conferring DNA hypermethylation to *Allene oxide synthase 1* (*AOS1*). Thus, OsSAPK10 increases rice immunity against *Xoo* via JA-mediated immunity (Hou et al., 2019).

## OsSnRK3/OsCIPK

Ca^2+^ signaling plays critical roles in numerous physiological processes, including plant growth, development, and stress responses (Kudla et al., 2018; Tang et al., 2020; Pirayesh et al., 2021; Ghosh et al., 2022; Koster et al., 2022). Since SnRK3s contain a NAF or FISL motif that interacts with CBL (**Figure 1**), they are known as CIPKs and are mainly involved in Ca^2+^ signaling. Kolukisaoglu et al. identified 30 *OsCIPKs* via sequence analysis (Kolukisaoglu et al., 2004). The expression of these genes is modulated in response to various abiotic stresses (Xiang et al., 2007; Kanwar et al., 2014). Overexpressing *OsCIPK3*, *OsCIPK12*, and *OsCIPK15* in rice enhanced tolerance to cold, drought, and salt, respectively (Xiang et al., 2007). Mutation of *OsCIPK31* resulted in hypersensitivity to ABA, salt, mannitol, and glucose, along with retarded germination and seedling growth (Piao et al., 2010). Although these findings imply that OsCIPKs play diverse roles in plant responses to different stimuli, their roles and regulatory mechanisms have remained elusive. Here, we focus on recent studies demonstrating the detailed functions of OsCIPKs.

## OsSnRK3 in plant response to abiotic stresses

**Figure 4:**
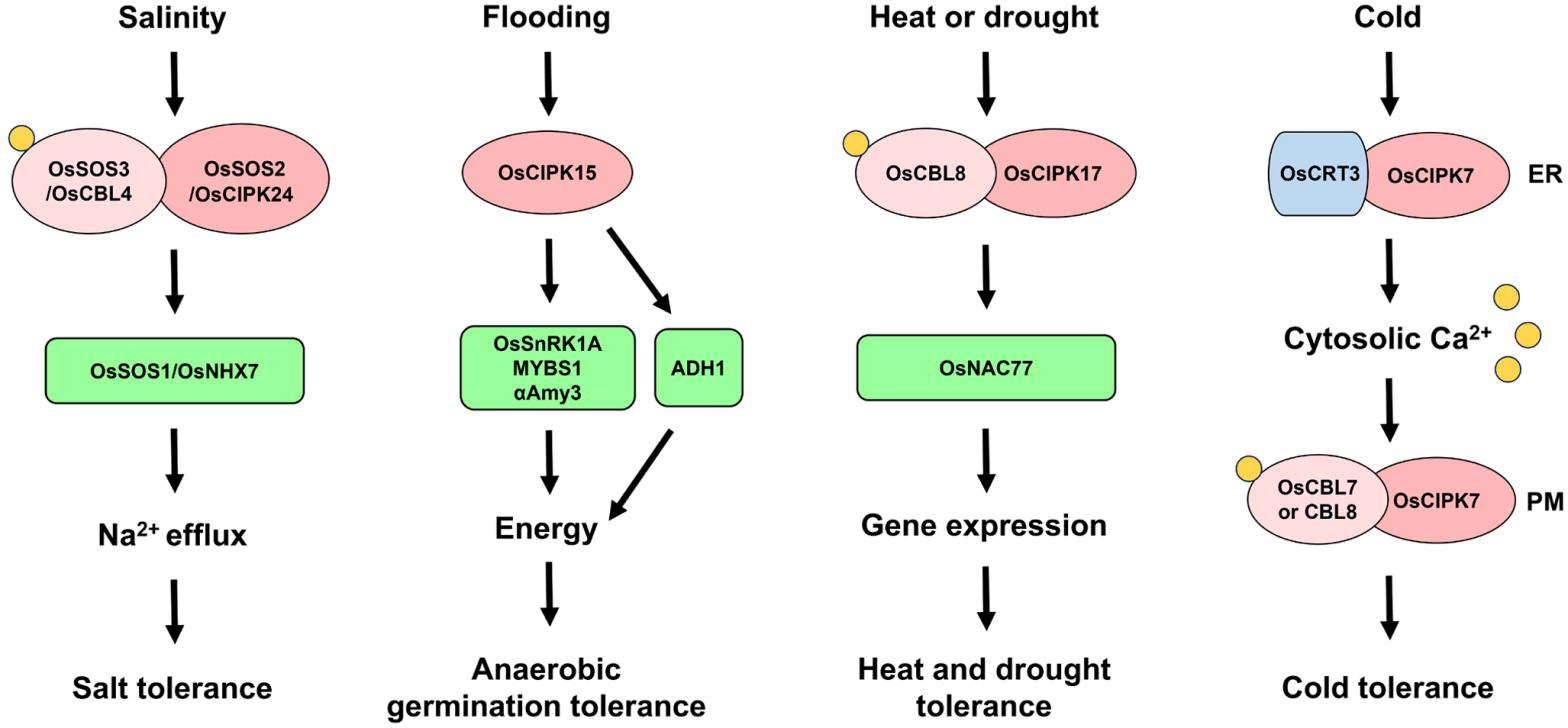
OsSnRK3 signaling cascades conferring abiotic stress tolerance. SNF1-related protein kinase 3 (SnRK3)/CBL-interacting protein kinase (CIPK) is key regulator of the Ca^2+^ signaling pathway in response to various abiotic stresses. Under salt-stress conditions, Salt overly sensitive 2 (OsSOS2)/OsCIPK24 forms complex with OsSOS3/OsCBL4 to recognize Ca^2+^. The complex then activates OsSOS1/NA^+^ and H^+^ antiporter 7 (OsNHX7) to maintain Na^+^ homeostasis (Martinez-Atienza et al., 2007). Flooding-induced sucrose starvation activates OsCIPK15 to regulate OsSnRK1A-MYBS1 module-induced starch mobilization via α-Amylase 3 (αAmy3), as well as ethanol fermentation via Alcohol dehydrogenase 1 (ADH1), resulting in energy production and anaerobic germination tolerance (Lee et al., 2009; Kudahettige et al., 2011). The OsCBL8-OsCIPK17 complex contributes to heat and drought tolerance by interacting with stress-associated proteins including OsNAC77, a transcription factor that induces the expression of genes related to heat and/or drought tolerance (Gao et al., 2022a). Under chilling conditions, Calreticulin 3 (OsCRT3) and OsCIPK7 undergo conformation changes in the endoplasmic reticulum (ER), resulting in high cytosolic Ca^2+^ levels and the formation of the OsCBL7-OsCIPK7 and OsCBL8-OsCIPK7 complexes, which activate Ca^2+^ signaling at the plasma membrane (PM) to enhance cold tolerance (Guo et al., 2023).

OsCIPKs are key regulators of the Ca^2+^ signal transduction pathway in response to various abiotic stresses. One of the best-known plant CIPKs is Salt overly sensitive 2 (SOS2), which is involved in the SOS signaling pathway conferring salt tolerance via ion homeostasis, as discussed below. The roles and signaling pathways of OsCIPK15 have been well demonstrated in rice. OsCIPK15 plays critical roles in plant tolerance to submergence and sugar starvation by regulating the OsSnRK1A-MYBS1 module. Rice has developed two different flooding tolerance mechanisms: the escape strategy and the quiescence strategy (Voesenek and Bailey-Serres, 2009). Anaerobic germination tolerance is the ability for germination and rapid coleoptile elongation with delayed radicle development in order to escape submergence conditions (Kuroha and Ashikari, 2020). The starvation-induced SnRK1A-MYBS1 module is important for regulating the expression of genes (e.g., *αAmy3*) involved in the mobilization of starch for germination and seedling development. Lee et al. showed that OsCIPK15 increases OsSnRK1A protein levels and the expression of α-amylase genes, resulting in anaerobic germination tolerance under flooding conditions (Lee et al., 2009). However, the flooding-induced expression of *OsCIPK15*, *MYBS1*, and *αAmy3* was significantly inhibited by sucrose (Kudahettige et al., 2011). Ethanol fermentation catalyzed by pyruvate decarboxylase (PDC) and alcohol dehydrogenase (ADH) is also important for anaerobic germination tolerance (Ismail et al., 2009; Takahashi et al., 2011). OsCIPK15 also upregulated *ADH1* expression under submergence, indicating that OsCIPK15 enhances flooding tolerance via not only starch mobilization but also ethanol fermentation (Lee et al., 2009). Therefore, OsCIPK15 confers anaerobic germination tolerance through activating both the OsSnRK1A-MYBS1-αAmy3 signaling pathway and the ADH1-mediated ethanol fermentation (**Figure 4**). However, OsCBL10 negatively regulates flooding tolerance by reducing OsCIPK15 protein stability and its downstream signaling pathways (Ye et al., 2018). OsCBL10 does not interact with OsCIPK15, suggesting that OsCBL10 affects OsCIPK15 via interactions with other unknown proteins. In rice variety FR13A, which contains the *Submergence 1A-1* (*SUB1A-1*) gene conferring the quiescence strategy, OsCIPK15-OsSnRK1A-MYBS1-αAmy3 signaling was not activated by flooding, but *SUB1A* and *Alcohol dehydrogenase 2* (*ADH2*) were dramatically upregulated under these conditions (Kudahettige et al., 2011).

Recent studies have revealed the roles of OsCIPKs in plant responses to other abiotic stresses. OsCBL8 and OsCIPK17 increase tolerance to heat and drought but decrease seedling growth (Gao et al., 2022a). The formation of the OsCBL8-OsCIPK17 complex was confirmed in both yeast and plants. OsCIPK17 interacts with stress-associated proteins including OsNAC77, a transcription factor that activates the promoters of genes conferring heat and/or drought tolerance (**Figure 4**). Thus, the OsCBL8-OsCIPK17 complex is thought to activate various downstream components for multi-stress tolerance. Moreover, OsCIPK7 plays a central role in the cold sensing and signaling pathway. Cold stress induces *OsCIPK7* expression. The mutation of alanine to valine at residue 169 in the T-loop of OsCIPK7 resulted in enhanced chilling tolerance in rice due to a conformational change that improved its kinase activity (Zhang et al., 2019a). Calreticulin (CRT) is a Ca^2+^-binding ER resident protein involved in various processes in eukaryotic cells (Joshi et al., 2019). Guo et al. showed that the expression of *OsCRT3* was specifically promoted by cold stress and that OsCRT3 functions as a cold senser by activating OsCIPK7 (Guo et al., 2023). OsCRT3 interacts with OsCIPK7 in the ER, and the cold-mediated conformational change in this complex increases its binding affinity and the kinase activity of OsCIPK7, leading to elevated cytosolic Ca^2+^ levels, perhaps due to Ca^2+^ efflux from the ER (Guo et al., 2023). OsCIPK7 also interacts with OsCBL7 and OsCBL8 at the PM, suggesting that Ca^2+^-activated OsCBL7 and OsCBL8 interact with OsCIPK7 at the PM and regulate Ca^2+^ signaling (Guo et al., 2023). Therefore, the cold-induced Ca^2+^ signaling by OsCRT3-OsCIPK7 and OsCBLs-OsCIPK7 complexes enhances plant tolerance under low temperature (**Figure 4**).

## OsSnRK3 in plant response to biotic stresses

OsCIPKs are also involved in biotic stress responses. *OsCIPK14* and *OsCIPK15* are duplicated genes that share over 95% nucleotide sequence identity. Their transcript levels significantly increased in response to PAMPs (i.e., *Trichoderma viride*/Ethylene-inducing xylanase and N-acetylchitoheptaose) (Kurusu et al., 2010). OsCBL4 interacts with OsCIPK14 and OsCIPK15 via the NAF/FISL motif, conferring their autoinhibitory activity and activating these proteins in a Ca^2+^-dependent manner (Kurusu et al., 2010). Although no effect of OsCIPK14 and OsCIPK15 on immunity against *M. oryzae* was observed in adult rice plants, these proteins enhanced PAMP-induced defense responses in cultured rice cells (Kurusu et al., 2010). Comparative transcriptome analysis revealed that *OsCIPK14* transcript levels dramatically increased in response to *R. solani* in sheath-blight-resistant rice variety GD66 compared to the sheath-blight-susceptible variety Lemon (Liu et al., 2022). Moreover, overexpressing *OsCIPK15* enhanced rice immunity against *Xoo* and *Xoc*, but OsASR6 (Abscisic acid, stress ripening-induced 6) inhibited the expression of *OsCIPK15* as well as resistance to these pathogens (Guo et al., 2022). Thus, the authors suggested that pathogen-induced OsASR6 is a negative regulator of OsCIPK15 conferring broad-spectrum disease resistance in rice (Guo et al., 2022).

## OsSnRK3 in ion signaling pathways

Ion homeostasis and signaling are important for plant adaptation to stress conditions. In particular, SOS-signaling-mediated Na^+^ efflux is crucial for salt tolerance. Three key regulators are involved in SOS signaling: the sodium (Na^+^) and proton (H^+^) antiporter (NHX) and the CBL-CIPK complex. Their regulatory mechanisms are highly conserved in plants, as described in previous reviews (Ji et al., 2013; Ali et al., 2023). In Arabidopsis, high levels of cytoplasmic Na^+^ trigger a Ca^2+^ spike, which is recognized by SOS3/CBL4. The activated SOS3 interacts with SOS2/CIPK24 and recruits it to the PM. The CBL-CIPK complex then activates the PM Na^+^ and H^+^ exchanger SOS1/NHX7. Thus, ion homeostasis conferring salt tolerance can be maintained through Na^+^ efflux. Three key regulators of the SOS signaling pathway were also identified in the model monocot rice: OsSOS1/OsNHX7, OsSOS2/OsCIPK24, and OsSOS3/OsCBL4 (Martinez-Atienza et al., 2007; Kanwar et al., 2014; El Mahi et al., 2019). Ishikawa et al. also showed that the mutation in *OsSOS2* significantly reduced the levels of radiolabeled cesium in the grains of this mutant compared to wild-type cultivar Koshihikari (Ishikawa et al., 2017). These results suggest that the SOS signaling conferring salt tolerance through Na^+^ homeostasis is well-conserved in rice (**Figure 4**). However, the regulatory mechanism remains elusive.

OsCIPK is also involved in potassium (K^+^) signaling. The OsCBL1-OsCIPK23 complex modulates the rice inward-rectifier channel Arabidopsis K^+^ transporters (OsAKTs). Li et al. showed that OsAKT1 is the major K^+^ uptake component in rice roots (Li et al., 2014). *osakt1* mutation and *OsCIPK23* silencing resulted in low K^+^ uptake efficiency and K^+^ deficiency symptoms, and the interaction of OsCIPK23 with OsCBL1 is required for the recruitment of OsCIPK23 to the PM and the activation of the transmembrane protein OsAKT1 (Li et al., 2014). OsAKT2 functions as a K^+^ uptake channel, primarily in the vascular bundles of shoot tissues. The OsCBL1-OsCIPK23 complex decreased the inward movement of K^+^ by OsAKT2 but increased K^+^ efflux activity in oocytes (Huang et al., 2021b).

Furthermore, several OsCIPKs are associated with ammonium (NH_4_^+^) signaling. NH_4_^+^ and nitrate (NO_3_^–^) are major forms of nitrogen, and NH_4_^+^ is used as the main nitrogen source for rice grown in the paddy field. *OsCIPK8*, *OsCIPK9*, *OsCIPK14*, *OsCIPK15*, and *OsCIPK23* were upregulated in response NH_4_^+^, whereas *OsCIPK3* was downregulated (Xuan et al., 2019). Indeterminate domain 10 (OsIDD10), a key transcription factor involved in NH_4_^+^ signaling, upregulated the expression of five *OsCIPK* genes in an NH_4_^+^-dependent manner and directly activated the promoters of *OsCIPK9* and *OsCIPK14* (Xuan et al., 2019). The *oscipk9* and *oscipk23* mutants show high NH_4_^+^ sensitivity in terms of root growth, like the *osidd10* mutant, and overexpressing *OsCIPK9* rescued this phenotype in the *osidd10* mutant (Xuan et al., 2019). Thus, OsCIPK9 is thought to be a downstream regulator of OsIDD10 in the NH4+ signaling pathway. Finally, nitrogen utilization efficiency and the recruitment of beneficial microbial communities increased in an *OsCIPK2*-overexpressing isogenic rice line compared to the wild type (Khan et al., 2019).

## Conclusions

Members of the SnRK family play central roles as hubs of sophisticated signaling pathways related to energy status, ABA, Ca^2+^, and responses to environmental perturbations. Recent evidence reveals the detailed regulatory mechanisms of the SnRK signaling networks and the downstream and upstream components that confer valuable traits for crop improvement. In this era of rapid climate change, crop food security is an important issue directly related to human survival that must be addressed in the near future. In this review, we summarized current knowledge about the biological functions and signaling networks of OsSnRKs in rice, a staple crop of half the worldwide population. The OsSnRK signal transduction pathway represents a valuable target of biotechnology for stress resilience. Therefore, further exploration of this protein family and its application for crop improvement will help us overcome the environmental challenges threatening food security worldwide.

## Author contributions

SS: Conceptualization, Supervision, Visualization, Writing – original draft, Writing – review & editing. SRP: Funding acquisition, Project administration.
